# Supplementary material for: What does it take to learn the rules of RNA base pairing? A lot less than you may think
Source: bioRxiv. 2025 Aug 2:2025.07.31.668042. Preprint. [Version 1] doi: 10.1101/2025.07.31.668042 (PMC12324431; doi:10.1101/2025.07.31.668042)
Supplement: Supplement 1 [file media-1.gz › supplemental_material/src/rscape_v2.6.0.tar.gz!/rscape_v2.6.0/lib/infernal/Manuscripts/banded-cyk/figs/scatter.pdf]

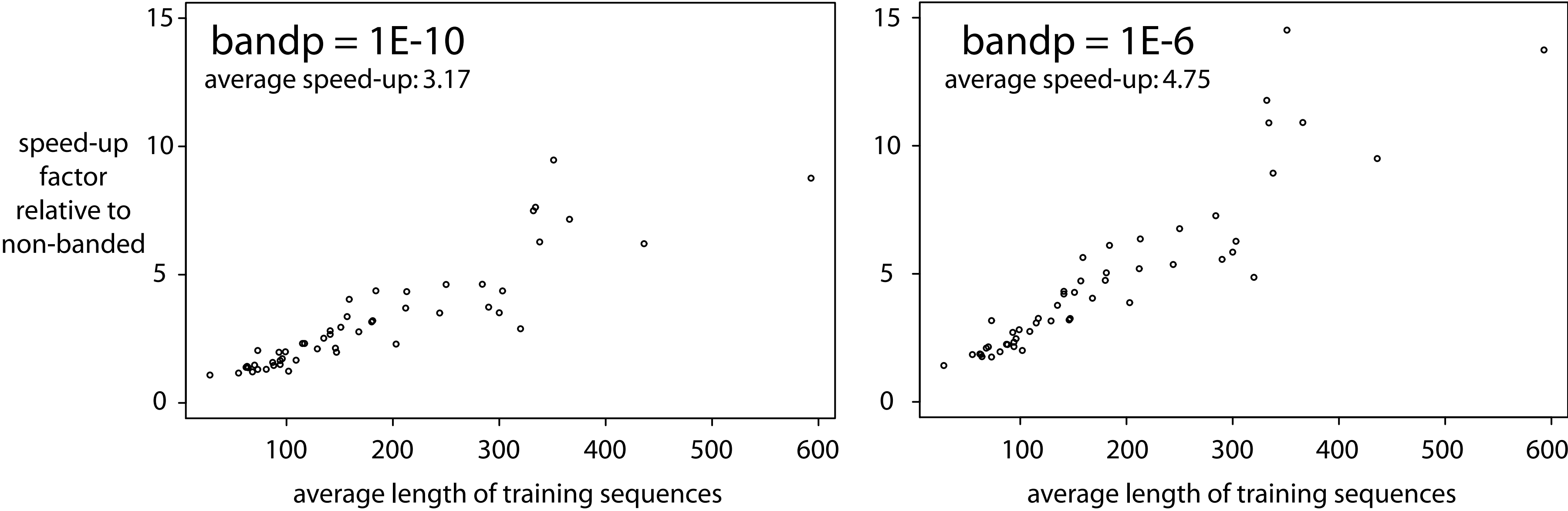

bandp = 1E-10
average speed-up: 3.17
bandp = 1E-6
average speed-up: 4.75
speed-up factor relative to non-banded
average length of training sequences
0
5
10
15
100
200
300
400
500
600
